# Dr. Samuel Stockton White

**Published:** 1880-01

**Authors:** 


### OBITUARY.

---

*Dr. Samuel Stockton White.*—It is with sincere regret that we announce the death of one, who has not only been closely identified with American Dentistry, but also with the Dentistry of the entire civilized world for many years, and to whose untiring energy, generous disposition, and business qualifications many of the most important improvements and inventions of our day and generation are due.

The name of "S. S. White" has long been a familiar one to dental practitioners wherever located, and almost numberless times, by his devotion to the interests of our science, and by timely assistance to those struggling to obtain a knowledge of our profession and the means whereby to practice it, has Dr. Samuel S. White merited the gratitude and earned the respect due to a benefactor of our race, and the friend of a liberal profession.

His splendid and successful achievements as a business man, and his character and reputation as a citizen afford a remarkable example, and one worthy of imitation, of what energy, pure motives and untiring perseverance may accomplish.

Dr. White left home, in company with a son and nephew, in November last, to travel for a time in Europe for the benefit of his health. They had been in Paris but a few days till he was taken suddenly ill, and this was followed within forty-eight hours with the news of his death, which occurred December 30th, 1879. Congestion of the brain was the cause.

We are indebted to his life-long friend, Dr. Wm. B. Atkinson, of New York, and to his brother, Dr. James W. White, of Philadelphia, for the following sketch of his life, which appeared in the Philadelphia *Times*, December, 31st, 1879:

"He was born in Hulmeville, Bucks county, Pennsylvania, in 1822, the son of William R. and Mary White, plain people, with little in the way of worldly possessions, but of eminent respectability. His father died when he was eight years old. The mother, left on her own resources, moved from their little village to Burlington, New Jersey, where she opened a candy store. The son remained with her, helping her in the business until he was fourteen years of age, when he came to Philadelphia and entered as an apprentice the establishment, on Vine street, of his uncle, S. Wesley Stockton, dentist and manufacturer of artificial teeth. That was forty-three years ago. Samuel Stockton White learned his trade, and as soon as he came of age began to practice dentistry in his uncle's office in addition to superintending his manufactory. Shortly afterward he removed to Eighth street, above Race, and went into practice for himself. There he remained till 1845. In that year he took in two partners—Asahel Jones, of New York, and John R. McCurdy, of Philadelphia. The manufacture of teeth began to make up a large part of their business, and in 1846 Mr. White seeing a wider field of operations relinquished his practice of dentistry and began to devote his time wholly to making teeth and dental instruments. They remained on Race street till 1848. Then they removed to a property on Arch street, below Sixth, which they had purchased and fitted up to accommodate their increasing business. Soon the business became the leading one of the country. White's teeth and White's dental instruments began to be known everywhere. Branch houses were established in New York, Boston and Chicago. Finally the partners sold out to their senior in the firm. McCurdy retired in 1859, Mr. White purchasing his interest, including real estate, for $140,000. Jones retired in 1861, his interest, including real estate, bringing exactly the same amount. In 1867 he removed again, and for the last time, from Arch street down to the massive building on the southeast corner of Twelfth and Chestnut streets, the lower portion of which is occupied by Bailey, Banks & Biddle's jewelry store. This is one of the largest buildings in the city, as well as among the costliest. It is five stories high, runs back to Sansom street and cost half a million dollars. Here the work of manufacturing teeth and dental instruments has been going on ever since. Over 300 hands are employed and 4,000,000 teeth are turned out annually.

But it was not alone to the manufacture of implements and appliances that his energies have been devoted. For thirty-three years he has exercised what may be termed a controlling influence in dental literature—first in his connection with the *Dental News Letter*, a quarterly journal, which was published for twelve years, and then succeeded by the *Dental Cosmos*, a monthly journal, now in its twenty-second volume.

Dr. White was, for many years, a member of the Arch Street M. E. Church. He was also a member of the Union League, the Reform Club, the Franklin Institute, the American Association for the Advancement of Science, the United States Board of Trade, the Pennsylvania Association of Dental Surgeons and of many other business, social and benevolent organizations. He dies worth about one and a half millions."

The Faculty of the Baltimore College of Dental Surgery invited the Dental Practitioners of Baltimore City to meet in the College Building on Tuesday Evening, January 6th, 1880, for the purpose of taking action relative to the death of Dr. White.

The meeting, which was largely attended, was called to order by Prof. F. J. S. Gorgas, when on motion, Dr. Henry H. Keech was elected President, and Dr. J. Emory Scott, Secretary.

Prof. Gorgas read a sketch of the life of the late Dr. S. S. White, and also a letter on the same subject from Dr. Wm. H. Atkinson, of New York. Eulogistic remarks were made by the President, Dr. Keech, Dr. A. W. Sweeney and others, and on motion a Committee was appointed to draft resolutions of respect to the memory of the deceased. The Committee, which consisted of Drs. F. J. S. Gorgas, T. S. Waters, and A. P. Gore, reported the following, which were unanimously adopted:

WHEREAS, The Dental Practitioners of Baltimore City having been called together to pay a tribute of respect to the memory of the late Dr. Samuel Stockton White, of Philadelphia, whose death may be regarded as both a professional and public loss; therefore be it

*Resolved*, That the Dental Practitioners of Baltimore City have learned with deep regret of the death of one who has been for many years closely identified with the Dental Profession, at that period of life when the mental powers are in their fullest vigor, when his honorable career as an enterprising business man had won for him a world-wide reputation, and whose personal qualities, as exemplified in every relation of life, had secured the warm attachment and high respect of a large circle of devoted friends.

*Resolved*, That the Dental Practitioners of Baltimore City fully recognize the obligations American Dentistry owes to the late Dr. S. S. White through the active agencies he has awakened for the elevation of the science, his devotion to its interests, and the zeal, energy and intelligence he displayed in useful inventions and dental literature.

*Resolved*, That this expression of appreciation of the worth of the late Dr. Samuel Stockton White, in the form of a copy of these resolutions, be transmitted to the bereaved family of the deceased, and that they also be published in the different Dental Journals.

Respectfully Submitted,

F. J. S. Gorgas,
T. S. Waters,
A. P. Gore,
*Committee.*

Professors Gorgas and Winder, on the part of the Faculty of the Baltimore College of Dental Surgery, and Dr. H. H. Keech, on the part of the Dental Practitioners of Baltimore City, were appointed to attend the funeral in Philadelphia.

